# Neurotoxicity of Prion Peptides Mimicking the Central Domain of the Cellular Prion Protein

**DOI:** 10.1371/journal.pone.0070881

**Published:** 2013-08-05

**Authors:** Silvia Vilches, Cristina Vergara, Oriol Nicolás, Gloria Sanclimens, Sandra Merino, Sonia Varón, Gerardo A. Acosta, Fernando Albericio, Miriam Royo, José A. Del Río, Rosalina Gavín

**Affiliations:** 1 Molecular and Cellular Neurobiotechnology, Institute for Bioengineering of Catalonia (IBEC), Barcelona, Spain; 2 Centro de Investigación Biomédica en Red de Enfermedades Neurodegenerativas (CIBERNED), Barcelona, Spain; 3 Department of Cell Biology, Faculty of Biology, University of Barcelona, Barcelona, Spain; 4 Combinatorial Chemistry Unit, Scientific Park of Barcelona, Barcelona, Spain; 5 Department of Physicochemistry, Faculty of Pharmacy, University of Barcelona, Barcelona, Spain; 6 Institute for Research in Biomedicine (IRB), Barcelona, Spain; 7 Networking Research Center on Bioengineering, Biomaterials and Nanomedicine (CIBER-BBN), Barcelona, Spain; 8 Department of Organic Chemistry, Faculty of Chemistry, University of Barcelona, Barcelona, Spain; Ohio State University, United States of America

## Abstract

The physiological functions of PrP^C^ remain enigmatic, but the central domain, comprising highly conserved regions of the protein may play an important role. Indeed, a large number of studies indicate that synthetic peptides containing residues 106–126 (CR) located in the central domain (CD, 95–133) of PrP^C^ are neurotoxic. The central domain comprises two chemically distinct subdomains, the charge cluster (CC, 95–110) and a hydrophobic region (HR, 112–133). The aim of the present study was to establish the individual cytotoxicity of CC, HR and CD. Our results show that only the CD peptide is neurotoxic. Biochemical, Transmission Electron Microscopy and Atomic Force Microscopy experiments demonstrated that the CD peptide is able to activate caspase-3 and disrupt the cell membrane, leading to cell death.

## Introduction

PrP^C^ is an endogenous GPI-anchored protein that is highly expressed in some neuronal and glial populations of the telencephalon (e.g., [1], [2], [3]). The N-terminal tail of PrP^C^ contains a signal sequence that promotes its intracellular trafficking to the Golgi network (e.g., [4]), an octarepeat region (OR) and a central domain (CD) (e.g., [5], [6]). The CD (residues 95–133) comprises two regions: the charged cluster (CC, residues 95–110) and the hydrophobic core (HR, residues 112–133), which makes up the first transmembrane domain (TM1) of PrP^C^ [7], [8]. Conversion of PrP^C^ to the β-sheet-enriched PrP^SC^ is responsible for prion pathology in transmissible spongiform diseases. Although the mechanisms that mediate this conformational change remain elusive (e.g., [9], [10]), it seems that some residues located in the HR are directly implicated in this process (e.g., [11]). In fact, based on the pioneering study of Forloni and coworkers [12], several researchers have used a synthetic PrP^C^ fragment of 21 residues of the CD domain (PrP_106–126_) as a model of prion neurotoxicity (e.g., [13], [14]), glial activation (e.g., [15], [16]) or phagocyte activation (e.g., [17]). However, the reported toxic properties of this peptide and the participation of the endogenous PrP^C^ in neurotoxicity differ between studies (e.g., [18], [19], [20], [21], [22], [23]). From a mechanistic point of view, some studies have reported that membrane modifications or the putative endocytosis of PrP_106–126_ mediate its neurotoxic effects [24], [25] in contrast to others [26], although it has also been reported that the peptide is able to modify membrane viscosity properties [27]. This is important if we take into account that membrane binding of PrP^C^ is required to induce neurotoxicity [28] (see [6], [29] for a review).

In the healthy nervous system non-amyloidogenic processing of proteins (e.g., amyloid precursor protein, APP) plays an important role in neuronal physiology (e.g., sAPPα as neurotrophin or long-term potentiation) [30], [31].

In fact, healthy PrP^C^ has been implicated in neurite extension and cell proliferation [32]. However, abnormal processing of these proteins leading to intermediate conformations of the protein (e.g., APP or PrP^C^) has been reported to produce cytotoxic species rather that the fibrillar amyloidogenic form (e.g., [23], [33]). Studies using chemically modified PrP_106–126_ have provided data on the physicochemical aspects of peptide toxicity *in vitro* [34], [35], supporting the idea that amyloid fibrils may not be the neurotoxic form of the prion (e.g., [36]). Indeed, the small oligomeric PrP species associated with the HR domain has been reported to be responsible for the highly characteristic thalamic pathology in Creutzfeldt-Jakob disease (CJD) [37].

In the present study, we used the full-length mouse CD peptide (residues 95–133) and its component regions CC and HR to determine participation in the neurodegenerative process associated with the CD. We show that the CD peptide, although not being refolded in a fibrillar manner, induces neuronal toxicity similar to that of PrP_106–126_. Surprisingly, electron microscopy revealed that the CD fragment presents protofibrillar structures in physiological solution, leading to progressive disorganization of phosphatidyl choline membranes as seen in atomic force microscopy, and promoting cell death independently of PrP^C^ expression in cultured cells. Despite the large number of studies reporting the neurotoxicity of different prion peptides, we indicate that the synthetic peptide comprising the CD domain is highly neurotoxic due to its inability to transform protofibrillar structures to mature fibrils.

## Materials and Methods

### Ethics Statement

All experimental procedures were performed in accordance with the guidelines of the Spanish Ministry of Science and Technology, following European Standards. The Animal Experimentation Ethics Committee (CEEA) of the University of Barcelona approved this study (document file number 115/11). Pregnant mice were sacrificed by cervical dislocation before removing the embryos. Prenatal and neonatal mice were euthanized by decapitation.

### Peptide Synthesis

Peptides mimicking the CC and HR (residues 95–110 and 112–133, respectively) of PrP^C^ were synthesized by Invitrogen (Carlsbad, USA, CA), and peptide PrP_106–126_ was purchased from Sigma Aldrich (Andover, UK). The CD-mimicking peptide (residues 95–133) was synthesized by the Combinatorial Chemistry Unit (UQC) of the Scientific Park of Barcelona (Barcelona, Spain) using Chemmatrix™-based (Matrix-Innovation™, Montreal, Quebec, Canada) solid phase synthesis.

The CD was synthesized on an Aminomethyl–ChemMatrix™ PEG resin [38] (Aminomethyl CM resin, 0.17 mmol, 0.62 mmol/g) and was washed before use as follows: MeOH (2×1 min), DMF (2×1 min), CH_2_Cl_2_ (3×1 min), TFA-CH_2_Cl_2_ (1:99) (3×1 min), DIEA-CH_2_Cl_2_ (1:19) (3×1 min) and CH_2_Cl_2_ (3×1 min). The AB linker (3-(4-hydroxymethylphenoxy) propionic acid) was incorporated with HATU-HOAt-DIEA (3:3:3:9). The first amino acid (Fmoc-Ser(OtBu)-OH) was introduced manually using DIPCDI:HOAt:DMAP (10:10:10:0.1) for 90 min, followed by an acetylation step. After elimination of the Fmoc group with piperidine:DMF (1:1, 20 min), elongation of the peptide was continued automatically on an ABI 433 A peptide synthesizer (Applied Biosystems, Foster City) using standard Fmoc chemistry and the FastMoc protocol using Fmoc-aa-HATU-HOAt-DIEA (10:10:10:30) as the coupling system. Cleavage was performed using TFA-TIS-EDT: H_2_O (94:1:2.5:2.5) for 90 min and the crude peptide was lyophilized under non-oxidative conditions. After lyophilisation, a disaggregating protocol [39] was applied. This protocol consisted of dissolving the crude peptide in hot TFA, removing the acid by evaporation, and dissolving the residue in HFIP. The crude peptide was characterized by analytical HPLC (30%) and MALDI-TOF (m/z calcd. 3977.6; m/z observed 3978.83 [M+H]^+^).

CD peptide purification was performed using an HPLC-MS semi-preparative system (Waters, Milford MA) with a reverse-phase Symmetry C_8_ column (30×100 mm, 5 µm) using a non-linear gradient (from 5 to 15% in 5 min and 15 to 35% in 20 min) of CH_3_CN (containing 0.1% TFA) and H_2_O (containing 0.1% TFA). Peptide detection was carried out via MS and UV absorption at 220 nm. Characterization of the final CD peptide was carried out by HPLC (*t_r_* = 10.7 min; 51%), HPLC-MS (m/z calcd. 3977.6; observed 1989.83 [M+2H]^+^/2, 1326.63 [M+3H]^+^/3, 995.26 [M+4H]^+^/4, 796.35 [M+5H]^+^/5, 663.86 [M+6H]^+^/6) and MALDI-TOF (m/z calcd. 3977.6; observed 3976.02 [M+H]^+^).

### Transmission Electron Microscopy (TEM) Procedures

Lyophilized peptides were dissolved directly in 0.1 M phosphate buffered saline (PBS) pH 7.4 (CC, CD) or in DMSO 98% (10X stock solution) and then 0.1 M PBS (HR), to obtain the appropriate concentrations (50–100 µM) for further experiments. For TEM, peptide solutions were fixed to Carbon-Forward-coated copper supports. After 0, 24 or 48 hours, negative staining was performed using a 2% PTA-based (phosphotungstic acid) stain (pH 7.4), after which samples were placed in silica-based desiccant for a minimum of 2 hours. Finally, we proceeded to TEM observation using a Leica microscope (Wetzlar, Germany) at the Electron Microscopy Service, University of Barcelona, Barcelona, Spain.

### Thioflavine T (ThT) Amyloidal Quantification Assay

ThT stock solution was prepared at 2.5 mM (dissolved in 10 mM phosphate buffer (potassium), 150 mM NaCl, pH 7.0) and preserved in single-aliquot form at −80°C. The ThT assay was performed by dissolving 50 µg of lyophilized peptide sample in 1 ml of freshly prepared ThT (final concentration 62.5 µM) followed by quantification using an absorbance/excitation spectrofluorometer LS-55 (Perkin-Elmer, Waltham, USA, MA). A peptide-free blank solution was used to measure residual ThT fluorescence. Non-refracting quartz cells (Hamamatsu Photonics, Hamamatsu, Japan) with a self-agitation system were employed to avoid fluorescence disturbance during experiments.

### Primary Neuronal Cultures and Peptide Treatments

*Prnp* knockout Zürich I mice (*Prnp^0/0^*) were purchased from the European Mouse Mutant Archive (EMMA, Monterotondo, Italy). *Prnp^0/0^* mice were backcrossed with C57BL6J mice for at least 10 generations to obtain 92–95% of the C57BL6J microsatellite markers (Charles River background analysis service), compared with the 46–48% found in Zürich I mice with a C57BL6J/129Sv mixed background [40]. Primary cortical cultures were prepared from E15.5–16.5 mouse embryo brains from heterozygous *Prnp^+/0^* parents as previously described (e.g. [19]). Briefly, cortical regions were isolated, trypsinized and treated with DNAse. After counting, healthy cells from each genotype were cultured in six-well culture plates (300,000 cells/well, 35 mm ø). Culture plates were from Nunc (Denmark), and culture media and supplements from Invitrogen (Carlsbad, USA, CA). After 5–7 days *in vitro* (DIV) and genotypic identification by PCR on tail-derived DNA, peptides were added to the culture media (see below).

Cerebellar granule neuron (CGN) cultures were prepared from 5-day-old pups (*Prnp^0/0^* and *Prnp^+/+^* genotypes) as described previously (e.g., [41]). Isolating and dissecting procedures, as well as culture media, were as described previously [42]. CGN were cultured at 120,000 cells per well (12-well plate, 12 mm ø coverslips) for 5 days prior to further treatment with peptides. Peptides were prepared as above and added to the cell cultures immediately after resuspension (or allowed to aggregate at room temperature for 24 h when required). The peptides were added to the culture media at concentrations ranging from 5 to 80 µM. In the case of HR peptide, the concentration of DMSO in the cell cultures was always below 0.5%.

### Cell Viability and Immunological Methods

Cell viability was assessed using a slightly modified propidium iodide (PI) uptake method, as described by Enguita et al. [43]. Propidium iodide fluorescence was measured in 24-well plates using an Infinite M200 PRO scanner (TECAN Group, Männedorf, Switzeland) with 530-nm excitation (25-nm band pass) and 645-nm (40-nm band pass) emission filters. Baseline fluorescence *F*_1_ was measured 1 h after addition of propidium iodide (30 µM) as an index of cell death not related to the treatment. Subsequently, fluorescence readings were taken at different times after the onset of the treatment. At the end of the experiment, the cells were permeabilized for 10 min with 500 µM digitonin at 37°C to obtain the maximum fluorescence corresponding to 100% of cell death (*F*_max_). The percentage of cell death was calculated as follows: % cell death = 100×(*F*_n_–*F*_1_)/(*F*_max_–*F*_1_), where *F*_n_ is the fluorescence at any given time. Cells were kept in the incubator between measurements.

In parallel, peptide-treated cells were scraped off in lysis buffer containing 1X protease inhibitor cocktail. Cell extracts were boiled in Laemmli sample buffer at 100°C for 5 min, followed by 10% SDS–PAGE electrophoresis, and then electrotransferred to nitrocellulose membranes for 6 h at 4°C, and processed for Western blotting using primary antibodies and detected by enhanced chemilluminescence using the ECL-plus kit from Amersham-Pharmacia Biotech, UK. Each nitrocellulose membrane was used to detect both protein levels of tubulin (Sigma Aldrich, Andover, UK) and cleaved caspase-3 (Cell Signaling, Beverly, MA, USA).

For immunochemistry, cells were fixed with 0.1 M phosphate buffered 4% paraformaldehyde (pH 7.4). α-Cleaved caspase-3 and α-neuronal-specific βIII-tubulin isoform (TUJ-1, Millipore) antibodies were employed to identify degenerating neurons. After mounting on Fluoromount™ (Vector Labs, Burlingame, USA), labelled cells were photodocumented using an Olympus BX61 fluorescence microscope equipped with a DX72 cooled camera. For quantification, the relative percentage of caspase-3-positive cells compared to TUJ-1-positive neurons was determined using Quantity One Image Software Analysis (Bio-Rad, Hercules, CA, USA).

### Atomic Force Microscopy (AFM) Procedures

Chloroform/methanol (50:50 (v/v)) stock solutions containing appropriate amounts of 1,2-Dimyristoyl-*sn*-glycero-3-phosphocholine (DMPC) were dried under a stream of oxygen-free N_2_. The resulting thin lipid film was then kept under high vacuum for several hours to ensure the absence of organic solvent traces. DMPC vesicles were obtained by hydration in an excess of resuspension buffer (RB) (10 mM Hepes, 150 mM NaCl, 20 mM CaCl_2_; pH 7.4). The spread of the planar lipidic membranes on mica was obtained using the vesicle fusion technique [44]. Briefly, aliquots (60 µL) of DMPC liposomes were pipetted onto freshly cleaved green *mica muscovita*, allowed to stabilize at 25°C (above the transition temperature of the phospholipid mixture) for 15 min, and then rinsed with imaging buffer (10 mM Hepes, 150 mM NaCl; pH 7.4). The probe was immediately immersed in the buffer. For all such experiments it was necessary to drift equilibrate and thermally stabilize the cantilever.

Peptide samples were prepared as described for culture/transmission electron microscopy. After resuspension, the peptide sample was injected into the AFM cell, and images were recorded in tapping mode with a commercial Digital Instruments (Santa Barbara, CA, USA) Nanoscope III AFM fitted with a 15 µm scanner (d-scanner). Standard Si_3_N_4_ tips, with a nominal force constant of 0.1 N/m (Digital Instruments), were used. Images were flattened using Nanoscope III software. The lipid-to-protein ratio (w/w, LPR) found to be appropriate for the performance of the experiments was 27∶0.5.

### SUVs Preparation and Permeability Assay of the Lipid Vesicles

The lipid was dissolved in chloroform/methanol (2∶1, v/v) and dried under a stream of oxygen-free N_2_. Lipidic vesicles were obtained by hydration of the resulting thin lipid film in 400 µl of a mixed solution of 12.5 mM ANTS, 45 mM DPX, 50 mM HEPES (pH 7.4) and 20 mM NaCl at a concentration of 1.0 mg/ml. Small unilamellar vesicles (SUVs) were prepared by sonication and the vesicles were added onto HiTrap™ (GE Healthcare, Buckinghamshire, UK) desalting column and eluted to 100 µM with 50 mM HEPES, 100 mM NaCl, pH 7,4. After 20 min of each peptide treatment, the fluorescence intensities were recorded with 355-nm excitation and 512-nm emission filters (Infinite M200 PRO scanner (TECAN Group, Männedorf, Switzeland)). The fluorescence intensity corresponding to 100% leakage was determined by adding Triton X-100 (2.5%, v/v) into the vesicles until the maximum intensity achieved [45].

### Statistical Analysis

All results are shown as mean ± SEM. One-way analysis of variance was used for statistical analysis of data using Statgraphics plus for Windows software version 5.1 (Statpint Technologies Inc., Warrenton, VA, USA). *p*<0.01 or *p*<0.05 were considered statistically significant.

## Results

### Characterization of the Peptides used in the Present Study

**Figure 1:**
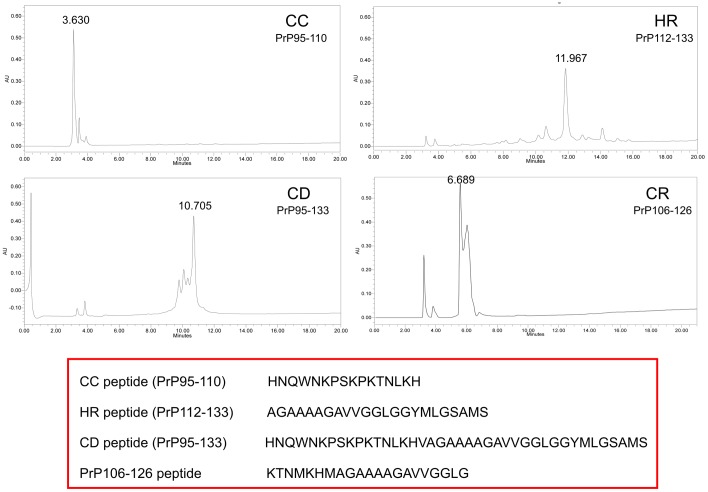
HPLC traces at 220 nm of CC, HR, CD and PrP_106-126_ peptides. HPLC conditions: Symmetry 300™ C_4_ column (4.6×250 mm, 5 µm) with a non-linear gradient of 0.1% aqueous TFA/0.1% TFA in CH_3_CN, from 95∶5 to 15∶85 over 5 min and 15∶85 to 40∶60 in 15 min at 60°C, flow rate of 1 mL/min. Note that the impurities are principally glycine deletions and are found in all four peptides. The extraordinary hydrophobic properties of the HR peptide in contrast with CC should be noted. The CD peptide clearly shows a mixture of biochemical properties of the HR and CC forms.

In order to decrease variability between peptide samples, three batches (#1, #2 and #3) of each peptide were purchased from Invitrogen or Sigma Aldrich or synthesized by UQC (see Materials and Methods section for details). Subsequently all peptides, regardless of their origin, were analysed via mass spectrometry prior to their use in the UQC. In terms of the quality of the fragment, the CC, HR and CD peptides presented similar profiles (Figure 1). Sample impurities likely corresponded to glycine-related deletions characteristic of the synthesis procedure, with similar levels observed in the three peptides. All batches presented equivalent mass spectra, showing no incongruence in terms of toxicity or aggregation. The results shown in Figure 1 were obtained using batch #2.

### TEM Analysis of Aggregative Properties of Peptides

**Figure 2:**
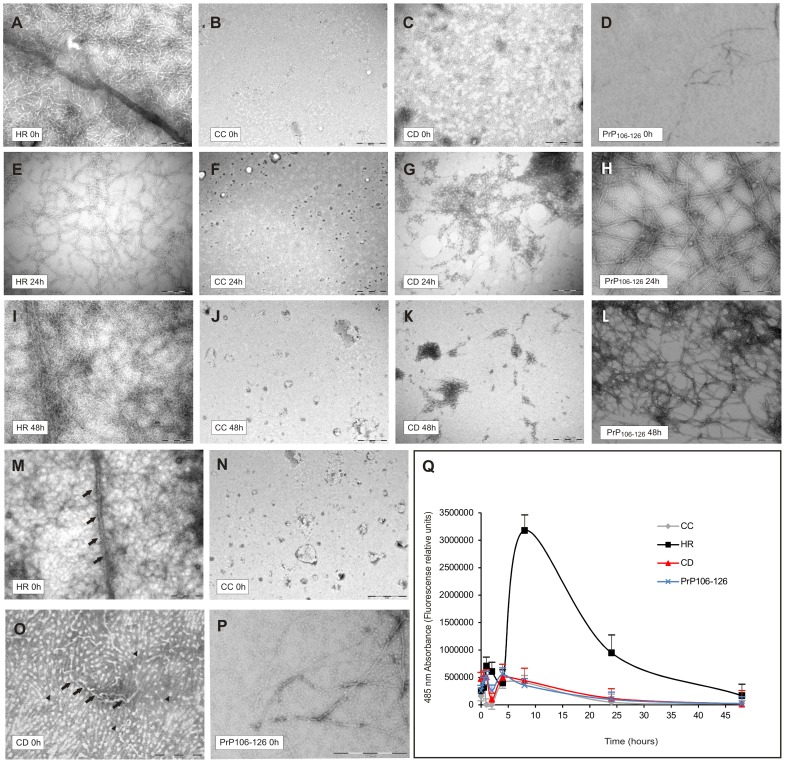
Kinetics of aggregation study of CC, HR, CD and PrP_106–126_ peptides through TEM and ThT fluorescence curves. A–L) TEM of the four peptides at 0, 24 and 48 hours post-dissolution. The HR peptide contains higher amounts of amyloid fibrils (A, E, I) than CC at all points (B, F, J). Note the progressive decrease in number of spherical structures over time in the CD peptide (C, G, K) and the coexistence of spherical and protofibrillar structures in the PrP_106–126_ peptide samples (D, H, L). Scale bars: A = 200 nm pertains to D–I, K–L; B = 500 nm pertains to C, J. M–P) TEM higher magnification of the four peptides just after dissolution. M) High TEM magnification of an amyloid fibril of HR peptide (black arrows). N) CC sample showing presence of no amyloid structure. O) High TEM magnification of spherical structures (black arrowhead). Black arrows indicate the presence of presumably intermediate or forming amyloid fibrils. P) PrP_106–126_ sample showing very small spherical structures together with protofibrillar aggregates. Scale Bars: M = 200 nm; N = 1 µm; O = 500 nm; P = 200 nm. Q) ThT assay showing ability of HR to form amyloidal structures. 50 µg of each lyophilized peptide sample were resuspended in 1 ml of freshly prepared ThT 62.5 µM. Note the strong increase in HR-derived fluorescence emission at 8 hours post-dissolution (∼3×10^6^ fluorescence relative units), and the progressive decrease up to 48 hours. The CD, CC and PrP_106–126_ peptides do not show any fluorescence peak over time. Three independent experiments were carried out with the three batches of each peptide, showing equal proportional results. The results presented correspond to the mean ± SEM of these independent assays.

In order to determine the aggregation kinetics of the synthesized peptides, TEM observations were performed at 100 µM of each peptide. As illustrated in Figure 2, TEM micrographs revealed the lack of fibrils in CC samples (Figures 2B, 2F and 2J). In contrast, TEM analysis of freshly prepared HR peptide samples showed the extensive presence of amyloid fibrils immediately after preparation (Figures 2A, 2E, and 2I), suggesting that this peptide might be already aggregated in the lyophilized state. In the case of PrP_106–126_ the micrographies initially showed very small spherical material together with some aggregates. After 24 h or 48 h of aggregation we observed some protofibrils and fibrils together with larger spherical structures. (Figures 2D, 2H and 2L). Surprisingly, we observed the presence of non-fibrillar structures in the substrate of the TEM sample corresponding to the CD peptide just after dissolution. The amount of these species decreased over time, suggesting the inability of this peptide to form mature fibrils (Figures 2C, 2G, and 2K). Higher magnification of these regions revealed the presence of round non-fibrillar structures similar to those observed in other neurodegenerative diseases [46], see Figure 2O. The progressive disappearance of the aforementioned species was observed in all CD batches analysed, so this effect is unlikely to be due to synthesis or sample variability. Figures 2 M, 2N and 2P shows TEM images with higher magnification of HR, CD and PrP_106–126_ peptides respectively.

### Thioflavine Assays Demonstrate that CD Peptide is not Amyloidogenic

We analysed aliquots of 50 µg/ml of CD, HR, CD and PrP_106–126_ peptides for the presence of amyloid fibrils at 0, 0.5, 1, 2, 4, 8, 12 and 24 hours after dissolution of the lyophilized state. ThT emits residual fluorescence in a fibril-free solution. However, ThT is able to bind amyloidal fibrils, showing a peak in fluorescence at 455/485 nm excitation/emission (see [47] for a review). In our experiments, the fluorescence levels of the four peptides were measured using a single ThT aliquot, and a blank sample from each aliquot was also used to test residual fluorescence from free ThT in each condition. For this reason, we can reject artefacts attributed to ThT aliquot variability or conservation state. Moreover, we can exclude solvent variability as neither DMSO nor PBS showed significant blank fluorescence levels.

As shown in Figure 2Q, the HR peptide presented the highest level of fluorescence emission at 8 hours post-dissolution (over 3×10^6^ fluorescence relative units). In maximal emission time the HR peptide showed a 7.2 and 7.79 fold increase with respect to the CD, CC and PrP_106–126_ peptides, respectively. After 24 hours, this peak in fluorescence declined slightly to ∼1×10^6^ units. However, there were no qualitative differences between 0 and 48 hours in TEM analysis (see Figures 2A, 2E and 2I) that would clearly explain the decrease in ThT. Between 0 and 8 hours, numerous intermediate structures with enhanced ThT-binding ability might be present in the sample. After 8 hours, the increase in mature fibrils might account for the decrease in ThT binding as also described for other peptides (e.g., Aβ [48]). Indeed, Goldslbury and coworkers point the higher increase in ThT fluorescence during the Aβ transition to form mature fibrils and show a similar decline in the fluorescence signal upon prolonged incubation of Aβ fibrils, suggesting that aged mature fibrils of this peptide react worse with ThT [48]. Since non-refracting quartz cells with a self-agitation system were employed to avoid fluorescence disturbance during ThT experiments, we can discard unspecific peptide adsorption to the cell walls or peptide precipitation to be responsible for this fluorescence decline. In contrast to HR, CC, CD and PrP_106–126_ peptides showed similar fluorescence to the blank sample in every emission measure. Lastly, only a few differences were found between different batches (#1, #2 and #3), as previously mentioned (see Material and Methods). Results in Figure 2Q shows the average between the three batches.

It is important to note the reported lack of ThT binding to PrP_106–126_ oligomers [49], while non-fibrillar oligomers of Aβ bind ThT, suggesting differing amounts of cross-β structure or poor accessibility to dye in the case of the PrP_106–126 _or CD peptides. Taking into account the poor understanding of ThT binding modes to amyloid fibrils, in a recent study M. Groenning propounded a model in which a cavity structure in the aggregated protein that may allow ThT to bind [50]. In fact, it has been described that some amyloid proteins display distinct patterns of fibrillation and ThT emission, that correlates with differences in the secondary peptide structures and the abundance of aggregates formed [51], [52]. In addition, we cannot rule out that specific conformation of formed fibrils (twisted *versus* non twisted fibrils, e.g., Figure 2M and Figure 2L) may also play a role in the observed results.

### CD Induces Neuronal Degeneration

**Figure 3:**
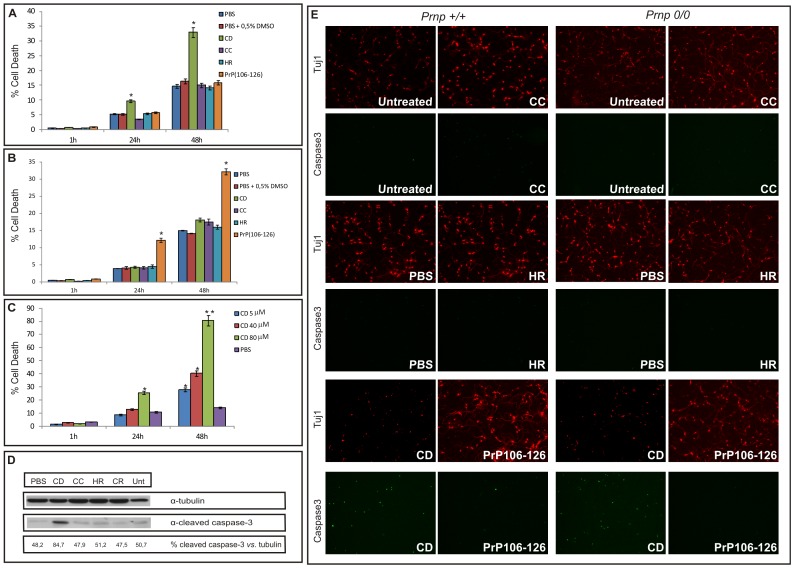
CD peptide induces apoptotic cell death independently of PrP^C^ expression. A) PI histogram showing the percentage of non-viable cortical neurons over time after stimulation with the peptides (40 µM) immediately after dissolution. Note the cytotoxicity exerted by CD peptide, in contrast to CC, HR, and PrP_106–126_, which are innocuous in the same conditions. B) The same PI histogram after stimulation with the peptides left to aggregate for 24 h. In this case only PrP_106–126_ shows relevant cytotoxicity. C) Dose-dependent cell death induced over time by freshly diluted CD peptide in cultured cortical neurons. Bars represent the mean ± SEM of three independent experiments (* *p*<0.05, ** *p*<0.01, *vs* vehicle-treated cells). D) Western blot showing the cleavage of caspase-3 induced by the 40 µM CD peptide. Tubulin was used as the loading control. E) Cleaved caspase-3 staining indicating apoptotic cell death induced by the CD peptide. Tuj1 (α-neuronal-specific βIII-tubulin isoform) was used as a marker for neurons. Note the independence from PrP^C^ expression.

We analysed cell viability after treatment with all four peptides using a quantitative measure of PI emission (see above for details). Each experiment was replicated three times with the different batches of peptides. The results indicate that only the CD peptide is able to induce an increase in neuronal death when used at 40 µM (∼10% and ∼35% at 24 and 48 hours, respectively) shortly after preparation (Figure 3A). When the CD peptide was allowed to aggregate for 24 hours before adding it to the cell cultures, no significant toxicity was observed (Figure 3B). These results suggest that cytotoxicity might be exerted by the unstable spherical species of the peptide detected by TEM (Figure 2C), which disappear over time after resuspension (Figures 2G and 2K). As expected from previous results (e.g., [19]), PrP_106–126_ showed significant toxicity when aggregated for 24 h after dissolution (Figure 3B), supporting the delayed appearance of toxic aggregated species in this peptide with time [53]. Lastly, we performed a time course with different concentrations of the CD peptide for different periods of incubation. The results showed that as low as 5 µM of CD peptide caused a 2-fold increase in cell death after 48 hours of treatment (Figure 3C). Higher concentrations induced higher rates of cell death, increasing drastically when 80 µM were applied (cell death rates: 20% and 80% at 24 and 48 hours, respectively, Figure 3C), indicating that cell death cause by CD peptide is dose-dependent. The results obtained in the PI fluorescence experiments were corroborated by the increase in cleaved caspase-3 detected by Western blotting after CD treatment. In contrast, the CC, HR and PrP_106–126_ peptides showed similar levels of cleaved caspase-3 to controls (both untreated and buffer incubated) at 40 µM (see Figure 3D) and 80 µM respectively (data not shown).

In order to determine whether death induced by the CD peptide was dependent on the expression of PrP^C^, cerebellar granule neurons from wild-type and *Prnp^0/0^* mice were treated with 100 µM of each peptide for 48 hours. The expression of PrP^C^ was determined by Western blot (Figure S1). Wild-type neurons treated with the CD peptide showed an increase in cleaved caspase-3 staining with respect to solvent-treated or CC-, HR- and PrP_106–126_-treated neurons. Similar staining was observed in *Prnp^0/0^* neurons (Figure 3E). Subsequent quantification of caspase-3-stained neurons revealed a significant increase in neuronal death in the CD (∼35% and ∼40% in *Prnp^+/+^* and *Prnp^0/0^*, respectively) compared to the PBS treatment (∼2.5% (*Prnp^+/+^*) and ∼3.5% (*Prnp^0/0^)*; Figure S2). As shown in Figure 3E, PrP^C^-deficient neurons seemed slightly more susceptible (although non-statistically significant) to peptide treatment than *Prnp^+/+^* neurons. This difference could be attribute to intrinsic neuronal *Prnp^0/0^* sensitivity as described previously [54], rather than to modulation of this death mechanism by PrP^C^.

### CD Peptides Disrupt Lipid Bilayers

**Figure 4:**
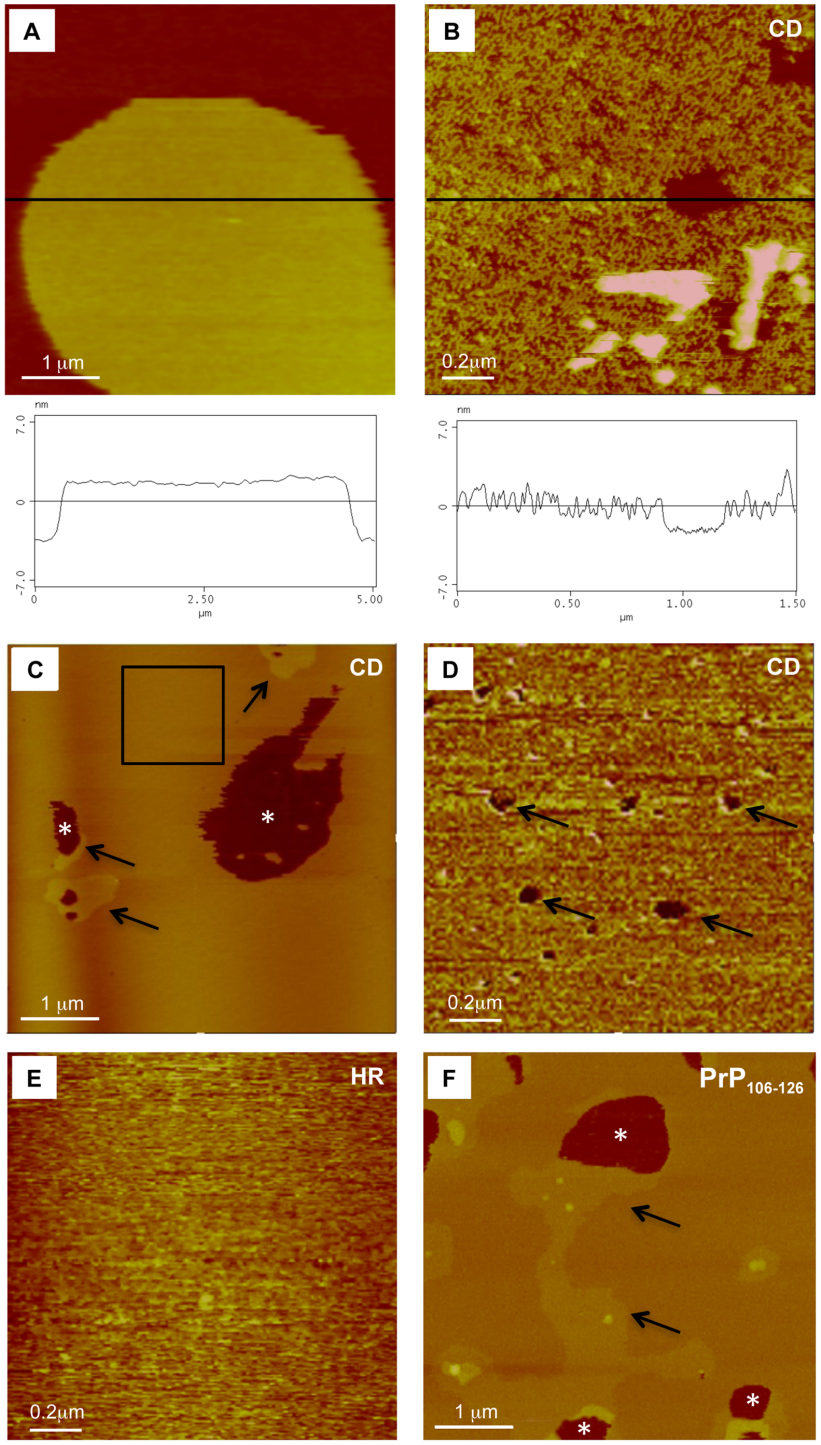
AFM analysis of DMPC membrane interaction with CD, HR and PrP_106–126_ peptides. Topographic images of control (A) and CD-treated artificial DMPC bilayer (B). Images were captured in tapping mode using 10 mM Hepes pH 7.40; 150 mM NaCl imaging buffer. Z = 15 nm. C) Topographic image of CD-treated bilayer obtained via tapping mode capture. Notice the appearance of small membrane disruptions (asterisk) and the increasing phase discontinuities (arrows). D) High power view of the boxed region in C illustrating the presence of both membrane disruptions and emerging phase discontinuities after CD treatment. E) High power view of HR-treated bilayer obtained via tapping mode capture as above. Notice the absence of the small membrane disruptions observed with the CD peptide. F) Topographic image of PrP_106–126_ -treated bilayer obtained via tapping mode capture using 24 hours resuspended peptide. Notice the appearance of membrane disruptions (asterisks) and phase discontinuities (arrows) in the membrane. Scale bars are indicated in each AFM image.

The interaction of several prion synthetic peptides with the plasma membrane has recently been analysed using AFM and other techniques (see e.g., PrP_110–136_ [55] or PrP_106–126_ [25], [26]). Our results indicate that the CD peptide is able to modify the stability of DMPC membranes, as observed in tapping mode scanning AFM analysis (Figure 4A–B, C–D). In contrast, the HR (Figure 4E), CC and PrP_106–126_ (not shown) peptides were unable to generate a similar disaggregation under the same conditions (without incubation time before AFM observation). In addition, parallel experiments showed that 24-hour-aggregated PrP_106–126_ was also able to induce DMPC membrane disorganization (Figure 4F)_._ AFM time course analysis, demonstrate that the disaggregation process of CD peptide implies the formation of several phase discontinuities in the DMPC bilayer with the increasing presence of holes surrounding these phase discontinuities (Figures 4C–D). This process is progressive, leading to complete disaggregation of the artificial membrane, and was observed in all CD batches in continuous cantilever tapping scanning of DMPC-treated lipid bilayers (Figure 4B). These results reinforce those presented previously indicating the low level of interaction between the HR region and the plasma membrane (see Discussion). Furthermore, they also suggest that the observed cytotoxic effects of CD might be associated with the formation of transient structures that are able to interact with the membrane.

**Figure 5:**
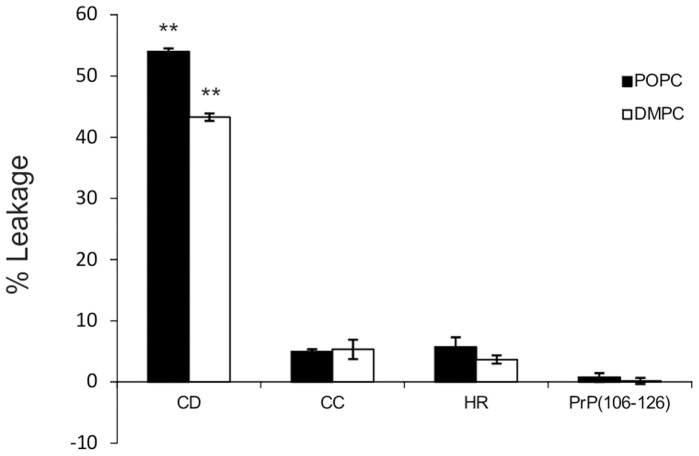
Effects of CC, HR, CD and PrP_106–126_ peptides on leakage of SUVs. Histogram showing the percentage of leakage of the vesicle (ANTS/DPX) of POPC or DMPC SUVs induced by the treatment with the different peptides at 40 µM. Bars represent the mean ± SEM of three independent experiments (** *p*<0.01 CD *vs* CC, HR and PrP_106–126_).

To further confirm the membrane perturbation induced by the CD peptide, ANTS-DPX leakage experiments were performed [45] (see also Materials and Methods for details). The results obtained show that, in contrast to CC, HR or PrP_106–126,_ the CD peptide increases permeability of POPC and DMPC SUVs just after dissolution (Figure 5). We observed a ∼11 and ∼9 fold increase in leakage in POPC and DMPC SUVs respectively at 40 µM peptide concentration (Figure 5).

## Discussion

Prion-mediated neurodegeneration requires the appearance of the abnormal misfolded form of the PrP^C^ within nervous tissue. In recent years, our knowledge concerning prion pathology has increased. We now know that the presence of prion aggregates lacking the GPI-anchor in the neural parenchyma does not exclusively condition the neurotoxic process, but GPI-anchored PrP^C^ is important in the amplification and spread of prion infectivity (e.g., [28], [56]). In fact, deletion experiments have shown that residues 108–124 of PrP^C^ participate in PrP^SC^ formation [57]. Several *in vitro* and *in vivo* studies have analysed prion neurotoxicity using peptides based on regions of its sequence mainly associated with the most conserved region of the protein (around the CR residues) (see [13], [58] for reviews and Table 1). PrP^C^ species lacking the N-terminal hydrophobic palindrome of PrP^C^; PrP (112-AGAAAAGA-119) or PrP (122Δ119) could not convert to the pathogenic prion [11]; and pioneer studies found that peptides containing residues 106–126 of the protein were neurotoxic ([12], see also [13], [58]). Following these findings, several studies have addressed questions such as i) whether the fibrillar form of the prion is the main form responsible for neurotoxicity (e.g., [23], [57], [59], [60]); ii) whether the cytotoxic effects of some of the peptide are mediated or enhanced by the endogenous cell expression levels of PrP^C^ (e.g., [21], [22], [61]) and iii) which part of the PrP^C^ region is responsible for peptide cytotoxicity (e.g., [46]) (see Table 1 for some examples). In the present study, we analysed the fibrillar properties of several peptides mimicking the CD of the PrP^C^. Although it is obvious that we cannot fully translate the present results to the full prion, some conclusions can be drawn.

**Table 1:** Reported properties of different synthetic prion peptides.

Peptide	Amyloidogenic properties	Toxicity	Membrane interaction	References
PrP_82–146_	No data	No data	Yes	[78]
PrP_89–106_	Yes	Yes	No	[27]
PrP_105–132_	Yes	Yes	Yes	[79]
PrP_106–126_	Yes	Yes	Yes	[13], [19]
PrP_112–126_	Yes	Yes	No data	[13]
PrP_113–120_	Yes	No	No data	[13]
PrP_113–134_	Yes	Yes	No data	[13]
PrP_118–135_	Yes	Yes	Yes	[61], [79], [80]
PrP_120–133_	Yes	Yes	Yes	[79], [80]
PrP_120–135_	Yes	Yes	Yes	[79], [80]
PrP_121–134_	No	No	No data	[13]
PrP_127–147_	Yes	Yes	No	[13], [27]

While fibrillar structures have long been considered the principal pathogenic agent in prion disease and other neurodegenerative disorders, there is growing evidence that amyloid oligomers or intermediate fibrillar structures are in fact the cytotoxic form that disrupts cell membranes through the formation of ion channels, pores or other protein–lipid complexes (reviewed in [46]). Although not fully comparable, in a study using the amyloid β (Aβ) peptide, Zhang and coworkers reported the higher neurotoxic action *in vitro* of non-fibrillar forms when compared with fibrillar structures [62]. In addition, a mutated form of the Aβ peptide with reduced fibrillar/aggregative potential showed increased toxicity [63]. Regarding the prion disease, a study of human samples showed that an increase in PrP oligomers correlated with disease severity in CJD [59]. In this scenario, we propose that the high degree of toxicity of the central domain peptide (CD) *versus* the other peptides assayed (Table 1) can be attributed to its inability to form fibrils and the large number of spherical oligomers observed shortly after resuspension. It is important to note that cell death is dependent on peptide concentration (see Figure 3) and time of aggregation, such that when the CD was allowed to aggregate for 24 h, toxicity declined due to the loss of toxic forms (see Figure 2).

Another example of a PrP^C^ peptide with *in vitro* and *in vivo* cytotoxic properties under non-fibrillar conditions is PrP_118–135_ [61]. Its properties are similar to those observed for the CD peptide. PrP_118–135_ mediates apoptosis independently of PrP^C^ expression [61]. Nevertheless, unlike the CD peptide, PrP_118–135 _has the capacity to form fibrils under determinate physical conditions without losing toxicity associated [61]. This is also the case for PrP_106–126_, which shows cytotoxic properties despite the capacity to form fibrils, possibly due to the mixture of non-fibrillar oligomers and amyloid fibrils or another species (see Figure 2) in the samples used (see also [49]). However, although conflicting [64], [65] another study reported that PrP_106–126_ was not cytotoxic [18]. Several *in vitro* studies have shown the high dynamics and reversibility in the equilibrium between monomer and protofibril formation of other amyloid proteins (e.g., Aβ [52]), and the coexistence of different species due to polymorphic fibril assembly pathways [66]. Regarding PrP_106–126_, the equilibrium between monomers and soluble oligomers, with an enrichment in secondary structures, is independent of concentration [34], but the fibrillar forms increase with time in a progressive manner [53] in parallel with the toxic intermediate structures. Taken together, the different described effects of PrP_106–126_ could be attributed to the particular experimental conditions in each study. In addition the presence of different species in the commercially available PrP_106–126 _with putatively different properties may also have had an impact in these studies. However, it is important to note that not all non-fibrillar aggregated species have toxic effects in cells [67] (see Table 1) explaining the lack of toxicity of PrP_106–126_ immediately after resuspension despite the annular structures seen in TEM images (Figure 2D, P) and points that distinct toxic oligomeric and/or annular intermediates may exist during amyloid formation [67], [68]. In this scenario PrP_106–126_ and CD peptide could present, at different aggregation times, distinct fibrillation intermediates that may share similar mechanisms of cytotoxicity, however the heterogeneous composition of intermediates of PrP_106–126 _at 24 h of aggregation increase the difficulty to study the toxic species in the sample.

Another aspect to consider is the relation between PrP^C^ expression levels and PrP peptide neurotoxicity. In previous studies, we found that the complete absence of PrP^C^ does not prevent death induced by high-dose exposure to aggregated PrP_106–126_ [19]. However, it has been reported that [21] the increased expression of PrP^C^ in cultured cells increased PrP_106–126_ neurotoxicity [22]. In fact, the absence or presence of PrP^C^, results in several changes in protein (e.g., [69]) or transcription levels ([70], [71], [72] which may pre-condition cultured cells to the effects of the synthetic peptides. Nevertheless, PrP^C^ is not required for the neurotoxic effects of some of the peptides. Thus, although the presence of PrP^C^ leads to increased binding of mimetic peptides or other amyloid proteins to the plasma membrane (e.g. [73] see also [74]), our results using *Prnp^0/0^* cells indicate that the presence of PrP^C^ is not mandatory for the neurotoxic effect. On the other hand, our data do not allow us to rule out the possibility that the increased presence of PrP^C^ in the plasma membrane may increase cell death by membrane alteration or other processes in the presence of the peptides.

Several studies have found that some PrP-mimetic peptides (e.g., PrP_106–126_ amide) are able to disrupt the lipid bilayer in AFM experiments [45] in a Ca^2+^-dependent manner [75]. In our AFM experiments a similar pattern of membrane disaggregation to that reported by Zhong and coworkers for PrP_106–126_ amide was observed only with the CD peptide (PrP_95–133_) and to some extent with aggregated PrP_106–126_ (Figure 4). This data was also corroborated by SUVs leakage experiments (Figure 5). These results may also be consistent with a recent report by Sauve et al. on PrP_110–136_ [55], in which resuspended PrP_110–136 _in water showed features of an unfolded protein in NMR experiments, and under physiological conditions had a higher affinity for dodecylphosphocholine micelles, being incorporated into the micelle in α-helical conformation [55]. Thus, the putative disaggregation of the membrane may lead to increased oxidative stress in treated cells and cell death (see [19]). On the other hand, the parallelism between intermediates in fibril formation in neurodegenerative diseases and pore-forming toxins (PFTs) (e.g., [33], [76]) is well documented, although the cellular processes involved still remain unknown. For example, it has been proposed that the fibrillar deposits are in fact a defence mechanism for sequestering deadly intermediate structures (reviewed in [77]). Given the difficulty of studying intermediate species in the fibrillar pathway due to their transitory nature, we propose that the CD peptide provides a tool with which to advance research on the physiological and pathological role of prion proteins.

## Supporting Information

Figure S1 **PrP^C^ expression in cultured cerebellar granule neurons (CGN).** Western blot analysis with 6H4 anti-PrP^C^ antibody of *Prnp^0/0^* and *Prnp^+/+^* CGN cultures after 3 DIV. Note the presence of the different PrP^C^ bands in the wild type and its absence from *Prnp^0/0^* cells.
(TIF)

Figure S2 **Quantification of micrographs showing CD-induced apoptosis in neurons (see** **Figure 3E** **).** Bars represent the mean ± SEM of three independent experiments (* *p*<0.05).
(TIF)
