# Supplementary figures and images for: Neurotoxicity of Prion Peptides Mimicking the Central Domain of the Cellular Prion Protein
Source: PLoS One. 2013 Aug 5;8(8):e70881. doi: 10.1371/journal.pone.0070881 (PMC3733940; doi:10.1371/journal.pone.0070881)

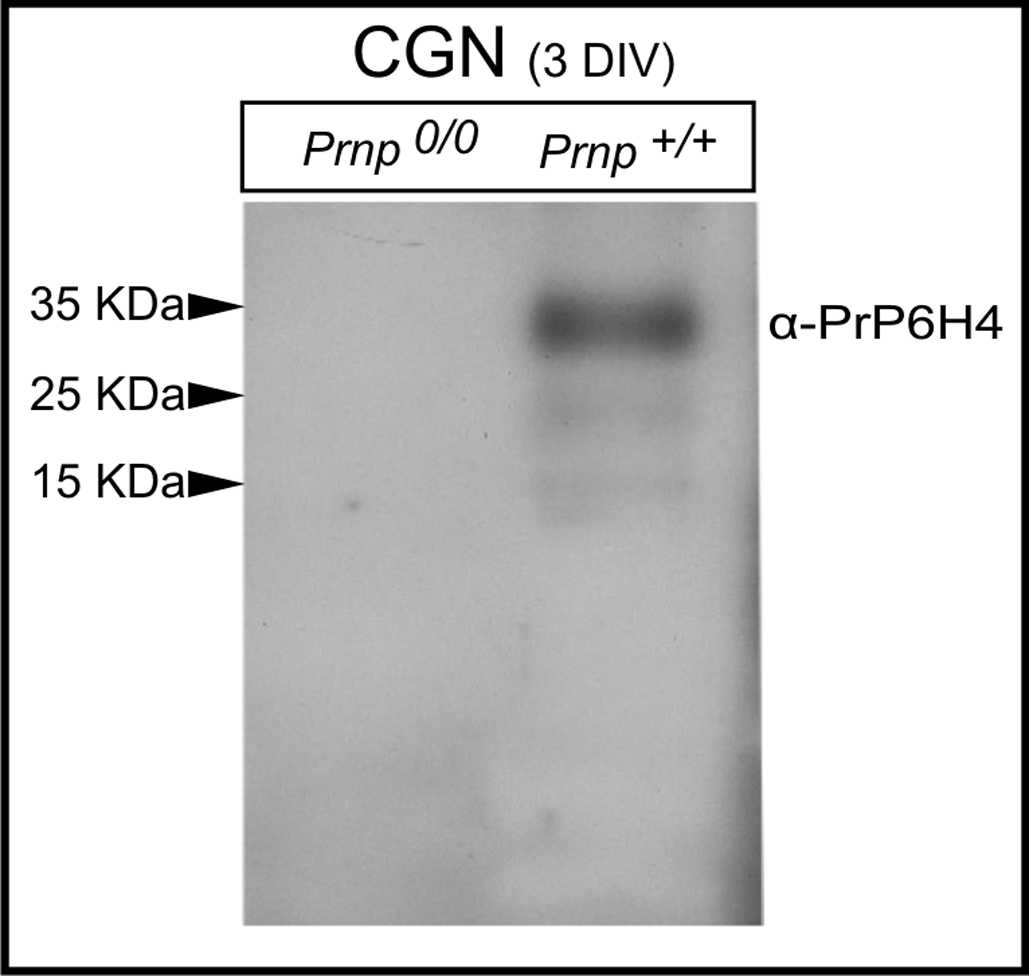

Supplement: Figure S1 — PrPC expression in cultured cerebellar granule neurons (CGN). Western blot analysis with 6H4 anti-PrPC antibody of Prnp0/0 and Prnp+/+ CGN cultures after 3 DIV. Note the presence of the different PrPC bands in the wild type and its absence from Prnp0/0 cells. (TIF) [file pone.0070881.s001.tif]

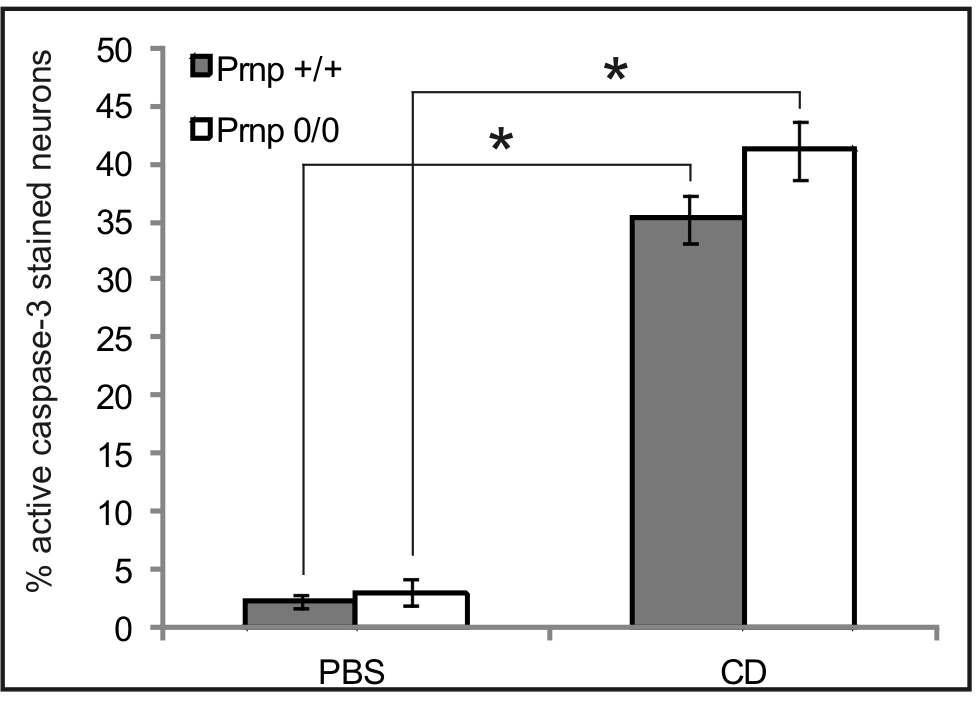

Supplement: Figure S2 — Quantification of micrographs showing CD-induced apoptosis in neurons (see Figure 3E ). Bars represent the mean ± SEM of three independent experiments (* p<0.05). (TIF) [file pone.0070881.s002.tif]
